# Characteristics and trends of acute poisoning in emergency departments: a single-center retrospective study in Zunyi, China (2021–2024)

**DOI:** 10.3389/fpubh.2026.1816492

**Published:** 2026-05-13

**Authors:** Xianjuan Gou, Xiaoli He, Na Li, Jing He, Ying Wang, Weiyan Tian

**Affiliations:** Department of Emergency, The Affiliated Hospital of Zunyi Medical University, Zunyi, China

**Keywords:** acute poisoning, chemical poisoning, drug poisoning, food poisoning, pesticide poisoning

## Abstract

**Background:**

Acute poisoning remains one of the leading causes of emergency department admissions, hospitalizations, and preventable mortality worldwide. To gain an in-depth understanding of the clinical characteristics of acute poisoning and provide a basis for formulating precise prevention and control strategies, this study aims to evaluate the clinical characteristics and trend changes of acute poisoning admitted to the emergency department of a hospital in Zunyi City from 2021 to 2024.

**Methods:**

This study retrospectively collected basic data on acute poisoning cases admitted to the emergency department of a tertiary general hospital in Zunyi City from January 1, 2021, to December 31, 2024. A descriptive analysis was performed on multiple characteristics, including age, gender, regional distribution, spring, and clinical outcomes.

**Results:**

A total of 3,146 acute poisoning cases were admitted, 2,789 valid cases were obtained, with an effective rate of 88.65%. The three most common poisoning types were drug poisoning (1,192 cases, 42.74%), pesticide poisoning (611 cases, 21.91%), and chemical poisoning (593 cases, 21.26%). Ages were mainly concentrated in the 10–20 and ≥ 60 years age groups, and there was a statistically significant differences in the age composition of different types of acute poisoning (*χ*^2^ = 365.831, *p* < 0.001). The number of female patients (1,604 cases) was higher than male patients (1,185 cases), and the gender difference was statistically significant (*χ*^2^ = 42.068, *p* < 0. 001). The incidence had obvious seasonal characteristics, with the highest number of cases in winter (830 cases, 29.76%), and the difference in seasonal distribution was statistically significant (*χ*^2^ = 473.396, *p* < 0. 001). There was no statistically significant difference in regional distribution (*χ*^2^ = 11.702, *p* = 0.470), but the number of cases in county areas was the highest (421 cases, 15.10%). Temporal trends showed that the number of drug poisoning cases increased significantly in 2022 while chemical poisoning cases decreased, and both pesticide and food poisoning cases peaked in 2023. In terms of clinical outcome, 91.43% (2,550 cases) of patients were cured or in stable condition at discharge, with pesticide poisoning having the highest mortality rate (2.6%), followed by drug poisoning (0.7%).

**Conclusion:**

Among the acute poisoning cases collected in this study, drug poisoning was the main type and showed an upward trend. The high-risk groups were the aged 10–20 and ≥60 years groups, and females, with the highest incidence in winter and no significant difference in regional distribution. Therefore, we should strengthen public education activities, enhance prevention and control for key populations, promote rational drug use, strengthen problem-solving strategies for proper drug storage, and improve the drug management and supervision system.

## 1 Introduction

Acute poisoning represents a significant global public health challenge, accounting for a substantial proportion of emergency department visits, hospital admissions, and preventable mortality worldwide. According to World Health Organization data, accidental poisoning resulted in 106,683 deaths and 6.3 million disability-adjusted life years lost in 2016 (1). Consequently, systematic surveillance of poisoning patterns and analysis of epidemiological characteristics are essential for developing effective prevention and control strategies.

Acute poisoning refers to a variety of adverse events induced by uptake of toxic substances, unintentionally or intentionally, within 24 h following exposure (2). According to the U. S. National Poison Data System (NPDS), an average of over 3.3 million poisoning cases are reported annually (3). Studies had reported that the post-hospitalization mortality rate is 0.45% (4). In contrast, acute poisoning ranks as the fifth leading cause of death in China, with its incidence increasing significantly, posing a serious threat to public health and social stability (5). Acute poisoning imposes a significant burden not only on individual health but also on society and the economy. One study revealed that environmental exposures and chemical mishandling contributed to 4.9 million deaths, accounting for 83% of the global disease burden (6). Another study estimated that the cost of post-hospitalization poisoning care and associated expenses for individuals aged 25 to 64 years reached approximately $8.125 million (7).

Previous studies indicated that the toxicological profile of acute poisoning varies across countries and regions, influenced by sociodemographic characteristics, economic development levels, and pesticide/ drug regulations. Pesticide poisoning is the most common type in low- and middle-income countries, whereas household products and drugs dominate in high-income countries (8). The American Association of Poison Control Centers (AAPCC) reported that the top five substance classes associated with human exposure were analgesics, cosmetics, household cleaning substances, sedatives/hypnotics/antipsychotics and foreign bodies (9). European studies indicate that the most frequent toxic exposures involve illicit drugs (primarily benzodiazepines), sedatives/hypnotics/antipsychotics, alcohol, and carbon monoxide (10).

This study aimed to investigate the epidemiological characteristics of acute poisoning cases admitted to the emergency department of a hospital in Zunyi City, Guizhou Province, over a four-year period, including demographic features, poison types, regional and seasonal distributions, and prognostic outcomes. The findings were compared with domestic and international literature to provide evidence for improving acute poisoning prevention and control strategies in Zunyi.

## 2 Materials and methods

### 2.1 Data sources

This study was a cross-sectional retrospective study. Data were obtained from the Hospital Information System (HIS) of the Affiliated Hospital of Zunyi Medical University. Medical records of all patients with a clinical diagnosis of “poisoning” treated in the emergency department were collected from January 1, 2021, to December 31, 2024. Diagnoses were established based on patients’ history, physical examination, and routine laboratory and toxicologic tests. The inclusion criteria were: (1) documented history of poisoning exposure; (2) clinical manifestations consistent with poisoning supported by characteristic laboratory abnormalities; and (3) confirmed diagnosis of acute poisoning. Cases of envenomation (animal bites/stings) were excluded.

Data were independently extracted and cross-verified by two researchers, with strict adherence to data management protocols throughout the study to ensure reliability. Cases with missing key variables were excluded, and only samples with complete data were included in subsequent statistical analyses. Variables with higher missing rates, such as region and clinical outcomes, were categorized as “unknown” without imputation. Outliers were identified using the interquartile range method and verified against original medical records; data entry errors were corrected, true extreme values were retained, and unverifiable records were excluded.

### 2.2 Definitions

Types of acute poisoning: we classified poisoning cases according to the Complete Book of Acute Poisoning (second edition) and NPDS annual reports (11, 12), categorizing toxic agents into five groups: chemicals, pesticides, drugs, and food products. For this study, “chemicals” were specifically defined as excluding pesticides, pharmaceuticals, biotoxins, warfare agents, and narcotics. Owing to its epidemiological significance, carbon monoxide was analyzed separately from other agents. The chemicals category was further divided into household chemicals (including cosmetics, cleaning products, disinfectants, automotive products, stationery, food additives, paints, coatings, adhesives, and construction materials) and industrial chemicals (including chemical raw materials, industrial additives, process intermediates, finished products, byproducts, waste materials, and unspecified chemical substances). Special classification considerations included: (1) mercury thermometers were classified as industrial chemicals due to difficulties in distinguishing elemental mercury sources; and (2) household insecticides being categorized as pesticides rather than household chemicals because their active ingredients are pesticidal compounds. Drug poisoning encompassed both misuse and abuse of Western pharmaceuticals and traditional Chinese medicines. Food poisoning referred specifically to oral exposure cases involving toxic plant/animal toxins or pathogens such as *Vibrio parahaemolyticus* and Salmonella.

Regional distribution was classified based on case origins into four categories: Main City, County, Other and Unknown.

The four seasons were categorized according to the meteorological seasons in China as follows: spring: March, April, May. Summer: June, July, August. Autumn: September, October, November. Winter: December, January, February.

The clinical outcome was observed with the patient’s discharge from the emergency department or hospital as the endpoint. Clinical outcomes were determined as follows: for acute poisoning patients who were discharged from the emergency department without hospital admission, their final clinical outcome was classified as unknown due to the lack of subsequent follow-up. For patients admitted to the hospital via the emergency department, clinical outcomes were categorized into four types based on symptoms, laboratory test results, and other indicators at discharge, which were retrieved from the hospital information system: (1) Recovery – complete resolution of symptoms with normalization of laboratory values; (2) Stable – symptom resolution with improved but not fully normalized test results; (3) Deterioration – worsening of both clinical presentation and laboratory parameters; and (4) Death – cases where clinical death was declared during hospitalization (excluding patients who discontinued treatment and expired after discharge) (13).

### 2.3 Ethical approval

The study protocol was reviewed and approved by the Ethics Committee of The Affiliated Hospital of Zunyi Medical University (Approval No. KLL-2024-581). The requirement for informed consent was waived due to the retrospective nature of the study.

### 2.4 Statistical analysis

Data entry and statistical analysis were performed using Excel and SPSS 22.0 software. For descriptive analysis, categorical data were presented as frequencies and percentages to describe the distribution of general demographic characteristics, poisoning types, regions, clinical outcomes, and other characteristics among patients with acute poisoning. Group comparisons were conducted using the chi-squared test (*χ*^2^). A two-tailed significance level of *α* = 0.05 was adopted, and *p* < 0.05 was considered statistically significant.

## 3 Results

### 3.1 Clinical characteristics of acute poisoning cases

A total of 3,146 acute poisoning cases were collected from the HIS system, with 2,789 cases deemed valid (effective rate of 88.65%). Among the valid cases, 654 cases (23.45%) were aged 10–20 years, 478 cases (17.14%) were aged ≥ 60 years. The remaining data are shown in Table 1.

**Table 1:** Clinical characteristics of acute poisoning cases (*n* = 2,789).

Variable	Frequency (n)	Percentage (%)
Age (year)		
<10	195	6.99
10–20	654	23.45
20–30	330	11.83
30–40	388	13.91
40–50	355	12.73
50–60	389	13.95
≥60	478	17.14
Gender		
Male	1,185	42.49
Female	1,604	57.51
Area		
Main Urban Area	370	13.27
Country	421	15.10
Other	276	9.90
Unknown	1722	63.54
Poisoning		
Chemical poisoning	127	4.55
Pesticide poisoning	611	21.91
Food poisoning	393	14.09
Drug poisoning	1,192	42.74
Carbon Monoxide Poisoning	466	16.71
Clinical Outcomes		
Stable	478	17.14
Recover	2072	74.29
Deterioration	79	2.83
Death	25	0.90
Unknown	135	4.84

### 3.2 Trends in the number of acute poisonings of different types

**Figure 1:**
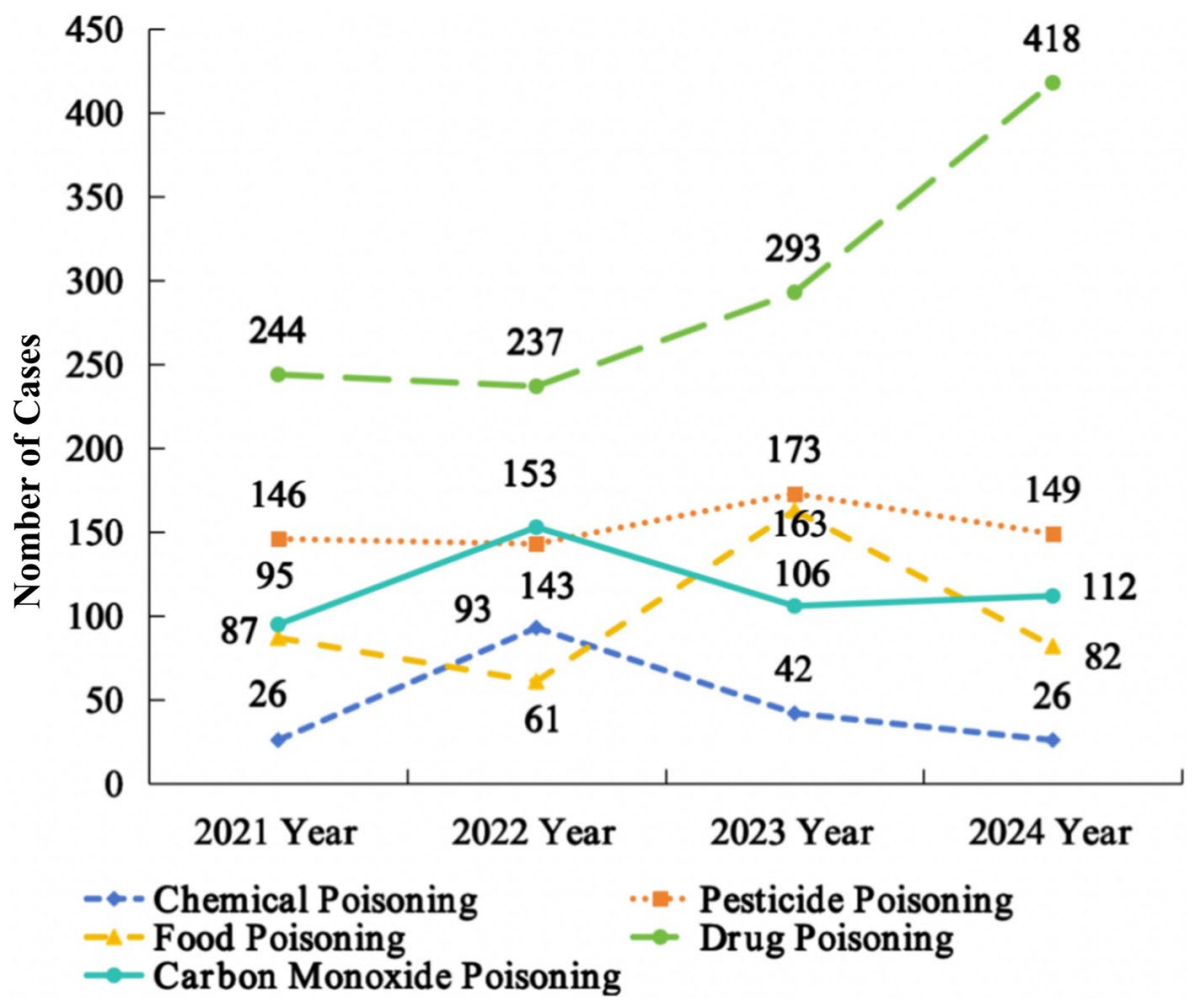
Trends in the number of acute poisonings of different types from 2021 to 2024.

Acute poisoning cases showed an increasing annual trend, with 598 cases in 2021 (598 cases), 627 in 2022, 777 in 2023, and 787 in 2024. Figure 1 demonstrates that drug poisoning cases increased while chemical poisoning cases decreased from 2022 to 2024. The peak incidence of pesticide poisoning (173 cases) and food poisoning (163 cases) occurred in 2023, whereas CO poisoning cases reached their highest number (153 cases) occurred in 2022.

### 3.3 Clinical characteristics of acute poisoning cases and comparative analysis of different poisoning types

As shown in Table 2, the distribution of acute poisoning types differed significantly with respect to age, gender, season, and clinical outcome (*p* < 0.05).

**Table 2:** Clinical characteristics of acute poisoning cases and comparative analysis of different poisoning types [*n* (%)].

Value	Chemical poisoning (*n* = 127)	Pesticide poisoning (*n* = 611)	Food poisoning (*n* = 393)	Drug poisoning (*n* = 1,192)	Carbon monoxide poisoning (*n* = 466)	*X* ^2^	*P*
Age (year)						365.831	<0.001
<10	17(13.4)	29(4.7)	36(9.2)	95(8.0)	18(3.9)		
10–20	11(8.7)	133(21.8)	32(8.1)	436(36.6)	42(9.0)		
20–30	15(11.8)	76(12.4)	47(12.0)	147(12.3)	45(9.7)		
30–40	20(15.7)	108(17.7)	69(17.6)	135(11.3)	56(12.0)		
40–50	28(22.0)	82(13.4)	79(20.1)	96(8.1)	70(15.0)		
50–60	22(17.3)	94(15.4)	63(16.0)	123(10.3)	87(18.7)		
≥60	14(11.0)	89(14.6)	67(17.0)	160(13.4)	148(31.8)		
Gender						42.068	<0.001
Male	66(52.0)	288(47.1)	187(47.6)	424(35.5)	220(47.2)		
Female	61(48.0)	323(52.9)	206(52.4)	768(64.4)	246(52.8)		
Area						11.702	0.470
Main Urban Area	12(9.4)	81(13.3)	62(15.8)	155(13.0)	60(12.9)		
County	22(17.3)	97(15.9)	67(17.0)	163(13.7)	72(15.5)		
Other	13(10.2)	58(9.5)	45(11.5)	111(9.3)	49(10.5)		
Unknown	80(63.0)	375(61.4)	219(55.7)	763(64.0)	285(61.2)		
Seasonality						473.396	<0.001
Spring	21(16.5)	169(27.7)	70(17.8)	352(29.5)	72(15.5)		
Summer	46(36.2)	165(27.0)	181(46.1)	298(25.0)	40(8.6)		
Autumn	26(20.5)	120(19.6)	90(22.9)	268(19.5)	41(8.8)		
Winter	34(26.8)	157(25.7)	52(13.2)	274(23.0)	313(67.2)		
Outcome						114.779	<0.001
Stable	22(17.3)	64(10.5)	80(20.4)	223(18.7)	89(19.1)		
Recover	97(76.4)	470(76.9)	289(73.5)	880(73.8)	336(72.1)		
Deterioration	4(3.1)	42(6.9)	5(1.3)	10(0.8)	18(3.9)		
Death	0(0.0)	16(2.6)	1(0.3)	8(0.7)	0(0.0)		
Unknown	4(3.1)	19(3.1)	18(4.6)	71(6.0)	23(4.9)		

## 4 Discussion

Acute poisoning is a major public health problem and a significant cause of emergency department visits (14). To achieve optimal treatment outcomes, poisoned patients require prompt evaluation. All acute poisoning cases are initially managed in the emergency department, where first-line treatment is administered. To elucidate the patterns and characteristics of acute poisoning in Zunyi, China, we investigated the demographic characteristics, regional and seasonal distributions, and referral characteristics of acute poisoning cases.

The results of this study showed that the total number of acute poisoning cases exhibited a continuous upward trend from 2021 to 2024, indicating that acute poisoning remains a persistent public health concern in emergency departments that cannot be ignored. This trend may be closely associated with the dynamic adjustments to COVID-19 prevention and control policies. During the period of epidemic prevention and control from 2021 to 2022, reduced social mobility and restricted access to toxic substances, coupled with some patients with mild symptoms being reluctant to seek timely medical care due to limited healthcare access, resulted in an underestimation of acute poisoning visits. With the optimization of prevention and control policies and the full resumption of social activities, patients’ access to medical care has been substantially improved, and the previously suppressed healthcare demand has been released. Combined with the increase in psychological stress events following the return to normal social order, these factors collectively contributed to the rebound and growth in acute poisoning visits.

In this study, there were 42.49% male patients and 57.51% female patients, with a significantly higher proportion of females than males (*p* < 0.001). These findings are consistent with reports by Rageh et al. (15) and Yang et al. (16), whereas Hurtado et al. (17) reported a male predominance (56.2%). Acute poisonings were most prevalent in the 10–20 – year age group (23.45%), followed by the ≥60 – year age group (17.14%). These demographic variations may be attributed to geographical, cultural, and socioeconomic factors.

Drug poisoning was the second most frequent type of poisoning in China. Our study found a higher proportion (42.74%) compared to Jiang’s study (10.63%) (18), which was consistent with Shazia’s study (19). According to the NPDS, drug poisoning incidence in the USA has risen rapidly since 2010, affecting all age groups, with psychotropic drugs being the predominant agents (20). Consistent with previous studies (21), our results revealed that drug poisoning was more common among women (64.5%). Existing evidence suggests this gender difference may be attributed to the substantial physiological and psychological effects of estrogen on emotional regulation and stress responses. When estrogen levels decline, negative emotions such as impulsivity, anxiety and depression may arise, which could precipitate self-harm or other extreme behaviors (22). In traditional Chinese families, women often bear heavy domestic burdens, including childcare, eldercare, and household responsibilities, which may lead to fatigue and greater susceptibility to mood disorders. In our study, drug poisoning cases were particularly prevalent in the 10–20 age group (36.6%), likely because this cohort includes both children and adolescents with distinct risk profiles. Children may accidentally ingest medications owning to improper storage or packaging that resembles food items. Adolescents are more prone to intentional drug misuse related to psychological factors such as depression and anxiety.

Pesticide poisoning was the second most common cause of acute poisoning in our study, accounting for 21.91% of the total cases. This proportion was lower than the 38.49% reported in Jiang’s study (18). The observed difference may reflect strengthened governmental efforts at various levels within our province to enhance poisoning prevention and control in recent years, including expanded public safety awareness campaigns and health promotion initiatives. The study revealed that pesticide poisoning cases predominantly affected females (52.9%), with the highest incidence occurring in the 10–20 age group (21.8%). The observed gender distribution was consistent with some previous studies (23) but inconsistent with others (24). This discrepancy may be attributable to multiple factors: (1) relatively underdeveloped economic conditions in the study region, leading to distinct gender-based occupational patterns; (2) certain biological and psychological traits more prevalent among females, including heightened emotional sensitivity and impulsivity; and (3) limited availability of mental health support services, which increases vulnerability to impulsive pesticide ingestion during periods of emotional distress (25–28). Consistent with Iranian research (24), pesticide poisoning peaked in spring (27.7%) and summer (27.0%). In China, these seasons correspond to periods of intensive agricultural production involving frequent application of herbicides and pesticides, which may account for the elevated incidence of poisoning.

The CO poisoning is one of the most common causes of accidental poisoning worldwide (29). In our study, CO poisoning accounted for 16.71% of all acute poisoning cases. This type of poisoning exhibited marked seasonal variation, with peak incidence in winter (67.17%) and a substantial decline during summer months—a pattern that is consistent with existing literature (30, 31). This temporal distribution likely reflects the association between CO poisoning risk and low ambient temperatures (31, 32). Globally, males account for nearly half of CO poisoning cases (33), which is potentially due to greater exposure risks from operating combustion equipment under poorly ventilated conditions or improper use. With cases concentrated mainly among those aged ≥ 60 years, which was significantly older than the ages reported in previous studies (31). This discrepancy may reflect region-specific exposure patterns in Zunyi, a third-tier city in southern China characterized by: (1) long, cold winters with low ambient temperatures; (2) widespread use of coal- and wood-fueled heating systems; and (3) limited public health infrastructure. The elevated poisoning risk stems from incomplete combustion of traditional fuels in poorly ventilated spaces, compounded by insufficient public awareness of CO hazards, suboptimal maintenance of heating equipment, and the lack of CO detectors in residential settings. We recommend implementing targeted interventions including community-based education on safe fuel utilization, stricter ventilation standards for heating systems, provision of affordable CO alarms, and enhanced emergency response training for local clinics.

In our study, food poisoning accounted for 14.09% of all acute poisoning cases, with higher incidence during summer and autumn. This pattern is attributable to climatic effects on the growth of foodborne pathogens. Park and Park (34) reported significant positive correlations between the incidence of food poisoning caused by Salmonella, *Vibrio parahaemolyticus*, and Campylobacter and environmental factors such as temperature, humidity, and rainfall. Zunyi’s unique ecological setting is characterized by mountainous terrain, extensive forest cover, and diverse vegetation. These features create favorable conditions for wild mushroom growth. The region’s long tradition of foraging for wild mushrooms increases the risk of accidental poisoning due to misidentification. The food poisoning mortality rate in our study was 0.3%, which was significantly lower than the historical rate previously reported in Zunyi City (35). This reduction may reflect recent public health interventions by provincial and municipal authorities, including enhanced public education campaigns on the dangers of toxic wild mushrooms, improved emergency response protocols, and community-based health promotion initiatives.

With economic development, the diversification and expanded production of industrial chemicals have heightened concerns about acute chemical poisoning. In our study, chemical poisoning accounted for 4.55% of all cases, with a male predominance (52.0%, male-to-female ratio = 1.08, 1). This finding is consistent with that reported by Zhang et al. (36). The most affected age group was 40–50 years, which reflects Zunyi’s socioeconomic context as a third-tier city. In this demographic, males typically serve as the primary breadwinners, with occupational exposures occurring through accidental ingestion during chemical handling and inhalation due to insufficient personal protective equipment. These risk factors are particularly prevalent in local manufacturing and small-scale industries (36).

The study demonstrated distinct clinical outcomes among acute poisoning patients: 74.29% were discharged after achieving clinical recovery, and 17.14% achieved clinical stabilization. Notably, 2.83% of patients left against medical advice despite exhibiting clinical deterioration, while the in-hospital mortality rate was 0.90%. This mortality rate was significantly lower than the value reported by Jiang (18). Pesticide poisoning was identified as the leading cause of death, accounting for 64% of all poisoning-related deaths. Previous studies have shown that suicidal poisoning constitutes a substantial proportion of fatalities. This may be attributed to the fact that patients with suicidal poisoning typically present with larger ingested doses, delayed hospital presentation, and a high incidence of multiple organ failure, resulting in significantly lower survival rates compared with accidental poisoning (37). Particularly notable was the higher rate of treatment discontinuation among patients with paraquat poisoning compared with other types of pesticide poisoning (18), which may reflect both the notoriously poor prognosis of paraquat poisoning and socioeconomic constraints associated with prolonged hospitalization. The mortality figures reported in this study may represent an underestimation, as we were unable to ascertain the final outcomes for 79 patients who left against medical advice while experiencing clinical deterioration and 135 patients lost to follow-up after discharge. This limitation suggests that the actual mortality rate could be higher than recorded, especially given the potentially severe prognosis of untreated acute poisoning cases.

The findings of this study provide important evidence for public health prevention and control of acute poisoning. Targeted interventions should be implemented for high-risk populations, including strengthening legislation and community education on safe household medication storage, and improving mental health screening and crisis intervention mechanisms for adolescents. Safety regulations for coal/gas heating equipment should be established, with targeted winter heating safety campaigns for older adult populations. For food poisoning control, enhanced surveillance and early warning systems for foodborne diseases during summer and autumn months are warranted.

## 5 Conclusion

Among the acute poisoning cases collected in this study, drug poisoning was the main type and showed an upward trend. The high-risk groups were the aged 10–20 and ≥60 years groups, and females, with the highest incidence in winter and no significant difference in regional distribution. Therefore, we should strengthen public education activities, enhance prevention and control for key populations, promote rational drug use, strengthen problem-solving strategies for proper drug storage, and improve the drug management and supervision system.

### 5.1 Limitation

This research has several important limitations that should be acknowledged. First, the hospital-based, single-center design and relatively small sample size may limit the generalizability of our findings. Future multicenter studies with larger cohorts would help validate these results. Second, although a lot of data were collected in our study, there was a lack of information regarding the causes and routes of poisoning, and a lot of “unknowns.” Therefore, this is among the things that motivates medical professionals to document specific information when treating poisoned patients. Third, clinical outcomes were determined only based on symptoms and laboratory indicators without standardized grading using PSS, lacking a unified quantitative tool, which may lead to subjective judgment and affect the accuracy and comparability of the results. Future studies should adopt standardized tools such as PSS to standardize outcome assessment and improve the objectivity and comparability of results.

## Data Availability

The original contributions presented in the study are included in the article/supplementary material, further inquiries can be directed to the corresponding author.
